# Colour duplex sonography of temporal arteries before decision for biopsy: a prospective study in 55 patients with suspected giant cell arteritis

**DOI:** 10.1186/ar2003

**Published:** 2006-07-19

**Authors:** Maria Karahaliou, George Vaiopoulos, Spiros Papaspyrou, Meletios A Kanakis, Konstantinos Revenas, Petros P Sfikakis

**Affiliations:** 1 Radiology Department, Laikon Hospital, 17 Agiou Thoma Street, Athens, 11527, Greece; 2 First Department of Propedeutic Medicine, Laikon Hospital, 17 Agiou Thoma Street, Athens, 11527, Greece

## Abstract

Although a temporal artery biopsy is the gold standard for the diagnosis of giant cell arteritis (GCA), there is considerable evidence that characteristic signs demonstrated by colour duplex sonography (CDS) of the temporal arteries may be of diagnostic importance. We aimed to test the hypothesis that CDS can replace biopsy in the algorithm for the approach to diagnose GCA. Bilateral CDS was performed in consecutive patients older than 50 years with clinically suspected GCA, as well as in 15 age- and gender-matched control subjects with diabetes mellitus and/or stroke and 15 healthy subjects, to assess flow parameters and the possible presence of a dark halo around the arterial lumen. Unilateral temporal artery biopsy was then performed in patients with suspected GCA, which was directed to a particular arterial segment in case a halo was detected in CDS. Final diagnoses, after completion of a 3-month follow-up in 55 patients, included GCA (*n *= 22), polymyalgia rheumatica (*n *= 12), polyarteritis nodosa, Wegener's, and Adamantiades-Behçet's diseases (*n *= 3), and neoplastic (*n *= 8) and infectious diseases (*n *= 10). A dark halo of variable size (0.7–2.0 mm) around the vessel lumen was evident at baseline CDS in 21 patients (in 12 and 9 uni- or bilaterally, respectively) but in none of the controls. The presence of unilateral halo alone yielded 82% sensitivity and 91% specificity for GCA, whereas the specificity reached 100% when halos were found bilaterally. Blood-flow abnormal parameters (temporal artery diameter, peak systolic blood-flow velocities, stenoses, occlusions) were common in GCA and non-GCA patients, as well as in healthy and atherosclerotic disease-control, elderly subjects. At follow-up CDS examinations performed at 2 and 4 weeks after initiation of corticosteroid treatment for GCA, halos disappeared in all 18 patients (9 and 9, respectively). We conclude that CDS, an inexpensive, non-invasive, and easy-to-perform method, allows a directional biopsy that has an increased probability to confirm the clinical diagnosis. Biopsy is not necessary in a substantial proportion of patients in whom bilateral halo signs can be found by CDS.

## Introduction

Giant cell arteritis (GCA), or temporal arteritis, is the most common form of systemic inflammatory vasculitis in adults. GCA is a panarteritis that insults older people almost exclusively. Because extracranial branches of the carotid artery are commonly affected, the most dreadful complication of the disease is blindness, which should be prevented by high index of suspicion and timely treatment with corticosteroids [1]. Given the lack of specificity both of the clinical and laboratory data [2], a temporal artery biopsy is always included in the algorithm for the approach to diagnose GCA [3]. For purposes of classification, the American College of Rheumatology (ACR) proposes that a patient with vasculitis is said to have GCA if three of the following five criteria are present: (a) age 50 years or above, (b) new onset of localized headache, (c) temporal artery tenderness on palpation or decreased pulsation, (d) erythrocyte sedimentation rate (ESR) of 50 mm/hour or more, and (e) histologic findings [4]. However, although a temporal artery biopsy is the diagnostic gold standard for this disease [5-7], biopsy results may be negative in 9% to 44% of patients with a clinical diagnosis of GCA [6-8].

Schmidt and colleagues were the first to propose ultrasonography as a quick, easy, and non-invasive test for identifying GCA, before biopsy [9-11]. These studies suggested that the presence of the halo sign (that is, a dark area around the vessel lumen probably due to arterial wall edema) is highly specific for GCA. This hypoechoic finding is circumferential and has to be demonstrated in two planes. Apart from the halo sign, stenoses and occlusions of the temporal arteries, frequently detected in acute disease, were recognized by Schimdt and co-authors as additional characteristic signs in patients suspected of having GCA [9,10]. The diagnostic value of colour duplex sonography (CDS), however, remains the subject of debate; Salvarani *et al*. have found that CDS does not improve the diagnostic accuracy of a careful clinical examination [12]. To further examine the diagnostic value of CDS in patients with suspected GCA, we designed a prospective study to specifically test the hypothesis that CDS can replace biopsy in the algorithm for the approach to diagnose GCA in the clinical setting.

## Materials and methods

### Protocol

Sixty consecutive patients aged 50 years or above who presented at the outpatient Rheumatology or Internal Medicine Clinics at Laikon Hospital (Athens, Greece) between 2000 and 2004 with clinical suspicion of GCA were prospectively studied. Provided that no other diagnosis could be established after a complete medical history, clinical examination, and routine laboratory examinations and chest X-ray, the inclusion criteria included at least one of the following: an ESR of 50 mm/hour or more, new headache onset, jaw claudication, fever, polymyalgia rheumatica, temporal artery tenderness, and recent visual impairment.

In parallel with the diagnostic work-up required for these patients, a baseline CDS of the temporal arteries was performed prior to the initiation of treatment. Unilateral biopsy of the temporal artery was subsequently performed within 1 to 5 days in 49 of the 55 patients who were included in the analysis. Biopsy was directed to a particular temporal artery segment in those patients in whom a halo sign in CDS was found. Irrespectively of the biopsy results, patients had to complete a 3-month follow-up to establish the final diagnosis, while CDS was repeated in 14 ± 1 day intervals after the initiation of treatment in those with abnormal baseline examination.

CDS of the temporal arteries was also performed in 15 age- and gender-matched healthy subjects (mean age 72, range 50–88 years), as well as in 15 patients with diabetes mellitus and/or stroke (mean age 73, range 51–92 years), who served as healthy and disease controls, respectively. Temporal artery biopsy was not performed in control subjects. The study protocol was ethically approved, and all subjects gave informed consent.

### Ultrasonography

All examinations were performed by at least one of three sonographers (MK, SP, and KR) using a Logic 500 MD, (General Electric Chicago, IL, USA), ultrasound system. Sonographers were experienced radiologists who were unaware of the presenting clinical findings of patients with suspected GCA at baseline. Studies in control subjects and follow-up examinations were performed in an unblinded manner. A high-resolution linear array (LA 39) 7- to 11-MHz transducer (General Electric) was used (length 45 mm). Superficial temporal arteries and frontal and parietal rami (to their distal points) were examined to assess temporal artery blood-flow parameters and the possible presence of a dark halo around the arterial lumen. System settings (most of them always uniform, some of them case-adjusted) were as follows: dynamic range 48 dB, colour gain: 20–26, type of colour gain V-6, wall filter less than 100 Hz, velocity scale 9–13 m/s, focus point position 5–7 mm (adjusted to vessel depth), zoom factor 1.5, signal volume length 1.

**Figure 1:**
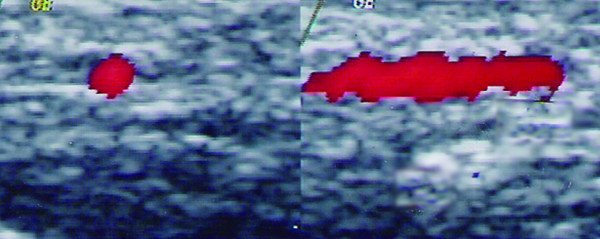
Normal common temporal artery. Demonstration of the left superficial temporal artery trunk by colour duplex sonography in a healthy person. Longitudinal (right panel) and transverse (left panel) planes.

**Figure 2:**
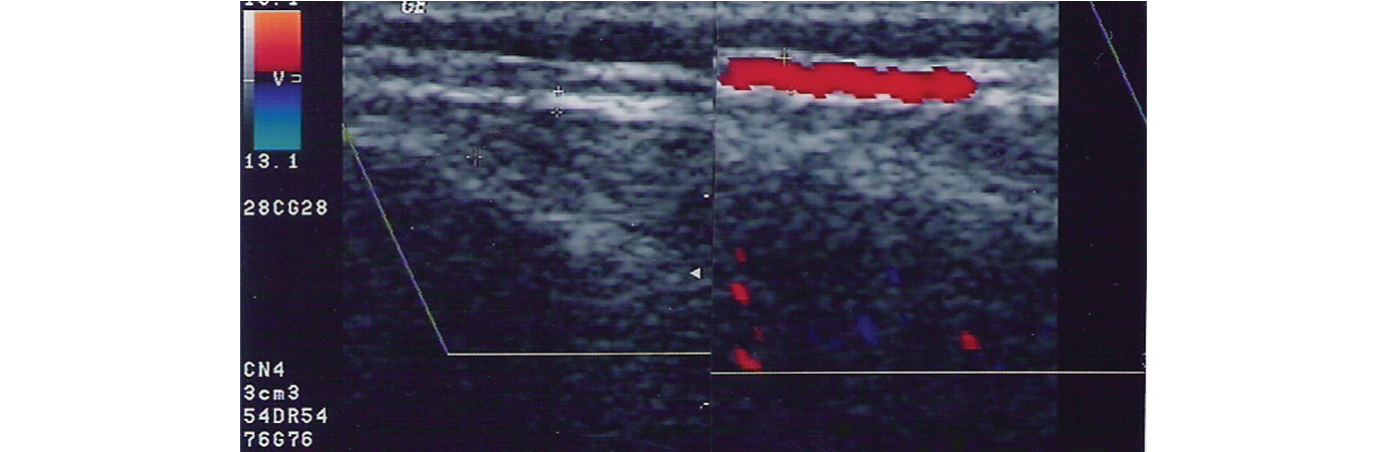
Parietal ramus of a normal temporal artery. Longitudinal view of the perfused lumen in colour duplex sonography; the bright area around the lumen represents the arterial wall plus the temporal fascia (right panel). With B-mode, the artery wall is still visible as two parallel bright lines (left panel).

Briefly, the principles of the method were as follows: By CDS, the anatomical route of the temporal artery could be presented in longitudinal and transverse view (Figure 1). The trunk of the superficial temporal artery could rather easily be found when the artery passes anterior to the tragus. From that point, the artery is bifurcated to frontal and parietal rami. Although the proximal segment of the artery trunk was not visible with B-mode (because it is not covered by fascia), the distal segment of the temporal artery trunk and both rami are embedded between the two layers of the fascia temporalis, which could be detected in B-mode as two parallel bright lines (Figure 2). Absent flow in the temporal artery was considered as arterial occlusion. In that case, detection of the temporal artery can be achieved by B-mode, except of the very proximal segment of the trunk. Segmental increase of blood-flow velocity, perhaps with waveforms indicating turbulence, was classified as stenosis in case it could not be attributed to other abnormalities (that is, artery kinking or atherosclerotic lesions). Stenosis was considered to be present in an area where (a) blood-flow velocity became at least double compared with the flow velocity recorded in the area right before, (b) there was local flow turbulence, and (c) there were low blood-flow velocities at the arterial segment right after. Measurements of the sagittal diameter of the systolic lumen, wall thickness, and peak systolic velocities were performed at the following three anatomical sites: (a) trunk: in front of the tragus, (b) parietal ramus: 15 mm distal of bifurcation, and (c) frontal ramus: 25 mm distal of bifurcation. When a stenosis was present at these points, measurements were performed 3–5 mm proximal. Finally, hypoechogenic ring areas, which appeared around the lumen of the temporal artery and could be detected in one or more sites unilaterally or bilaterally, were defined as halos. Measurements of the halo length and sagittal diameter at longitudinal and transverse level were also performed.

## Results

### Baseline characteristics and final diagnoses in patients with clinically suspected GCA

Of 60 consecutive patients with clinically suspected GCA who entered the study, five patients did not complete the 3-month follow-up and were excluded from the analysis. In the 55 patients, a final diagnosis was established and was then confirmed by follow-up and histology. Baseline clinical characteristics in GCA (mean age 70, range 52–90 years, 13 women) and non-GCA patients (mean age 74, range 50–91 years, 17 women) are shown in Table 1. ESR at 1 hour ranged from 40 to 129 (mean 88) and from 34 to 119 (mean 60) in GCA and non-GCA patients, respectively. Final diagnoses included GCA (*n *= 22), polymyalgia rheumatica (*n *= 12), polyarteritis nodosa, Wegener's, and Adamantiades-Behçet's disease (*n *= 3), and neoplastic (*n *= 8) and infectious diseases (*n *= 10) (Table 2). Considering the final diagnosis, an elevated ESR had a sensitivity of 86% but a low specificity of 9%. Both sensitivity and specificity of fever, new headache onset, and diffuse musculoskeletal pain were modest, whereas jaw claudication, temporal artery tenderness, and acute visual impairment had high specificity but low sensitivity for GCA diagnosis (Table 3).

### Diagnostic value of the halo sign in temporal artery CDS

**Figure 3:**
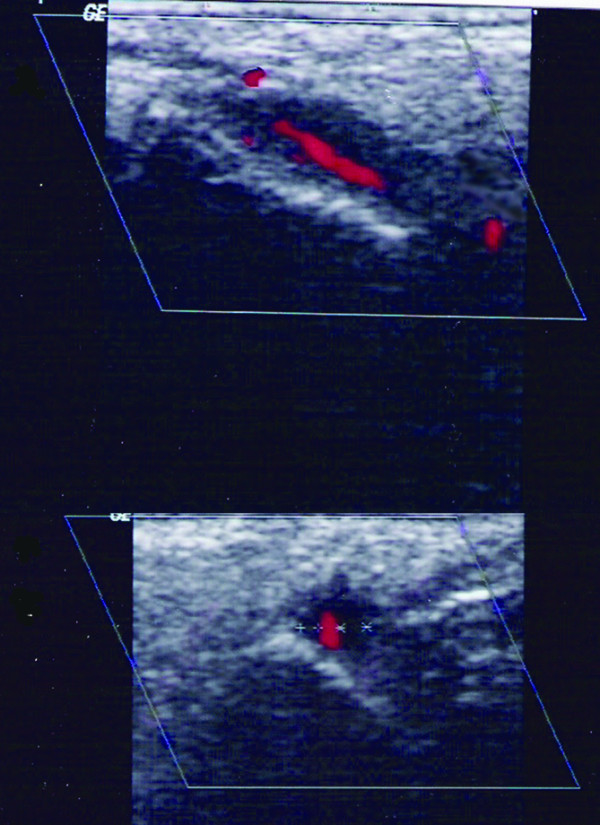
Halo sign in colour duplex sonography examination in a patient with giant cell arteritis. Hypoechoic area around the temporal artery trunk in longitudinal (upper panel) and transverse (lower panel) views.

In 21 of 55 patients with clinically suspected GCA, a concentric, hypoechogenic halo suggesting temporal artery wall inflammation was detected at baseline (Figure 3). Halos were detected at one or more sites of the artery. Although an inflammatory wall thickening was evident along the whole length of a particular branch in some cases, a segmental, patchy appearance of distinct, well-defined halos (length ranging between 5 and 15 mm) was evident in CDS. Halos' sagittal diameter ranged from 0.7 to 1.3 mm and in one case approached 2.0 mm. The halo sign was either a unilateral or a bilateral finding in 12 and 9 patients, respectively (Table 2). No halo could be demonstrated in any of the age- and gender-matched healthy control subjects or controls with diabetes mellitus and/or stroke (Table 2).

As shown in Table 2, the halo sign was present in 18 of 22 patients with GCA, and directional biopsies confirmed the diagnosis in all. In three additional cases (2 patients with infectious diseases and 1 patient with Wegener's granulomatosis; Table 2), a unilateral halo sign was found in CDS. In the first case, a vein neighbouring an artery was mistaken for a halo; the final diagnosis was a flu-like syndrome. In the second case, the final diagnosis was tuberculosis infection; biopsy results were negative for GCA in both patients. The third patient had a hypoechogenic halo of 0.6–1.1 mm at the right common superficial temporal artery and was finally diagnosed with Wegener's granulomatosis, a condition in which involvement of temporal arteries has been described [12-14]. A temporal artery biopsy, which albeit was not directed in this particular patient, was negative for vasculitis.

Of the four GCA patients without halo signs in CDS, a temporal artery biopsy (arterial segment ≈ 3 cm) confirmed the diagnosis in three; the last patient had a negative biopsy, but follow-up and the successful introduction of corticosteroid treatment made other diagnoses unlikely.

As shown in Table 3, the presence of a halo sign alone yielded 82% sensitivity (95% confidence intervals [CIs]: 71.85%–92.2%) and 91% specificity (95% CIs: 83.4%–98.6%) for the diagnosis of GCA, whereas the positive and negative predictive values were 86% and 88%, respectively. Although the presence of bilateral halo signs was not common (sensitivity 41%, 95% CIs: 28%–59%), the specificity of this finding reached 100%. Histology results were obtained in all 22 GCA patients and in 27 of 33 non-GCA patients, but because all 18 positive biopsies in GCA patients were directed to a particular arterial segment after obtaining the CDS results, the true value of the temporal artery biopsy alone for the diagnosis of GCA could not be estimated in our patients.

### Temporal artery flow abnormalities

**Figure 4:**
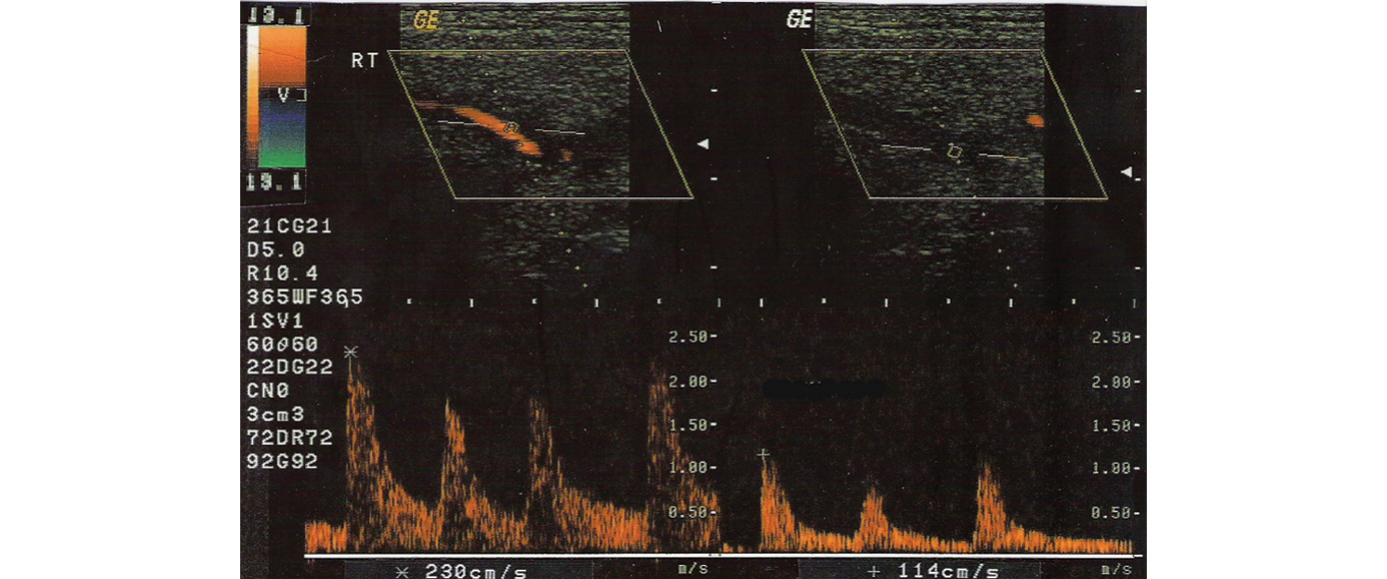
Doppler waveform at temporal artery trunk indicative of stenosis in a patient without giant cell arteritis. The peak systolic velocity (left panel) becomes at least double compared with the rate recorded in the area proximal to the stenosis (right panel).

As shown in Table 2, various flow abnormalities, mainly segmental stenoses (Figure 4), were detected by CDS in 18 of 55 patients with clinically suspected GCA, as well as in 11 of 30 control subjects, indicating that flow abnormalities of the temporal arteries could not discriminate the patients with GCA. Among the patients with suspected GCA, one had occlusion of the frontal ramus. Flow velocity was lower at halo sites in many patients. However, low flow velocities were also evident in patients without GCA as well as in control elderly subjects with or without diabetes mellitus and/or stroke. Mean values of parameters such as lumen systolic diameter and maximum systolic velocity, measured at three anatomical points as described in Materials and methods, were not significantly different between patients with suspected GCA and age- and gender-matched controls (data not shown).

### Follow-up CDS examinations

Follow-up results obtained 14 ± 1 days from baseline in those patients with negative CDS examination remained unremarkable. Notably, halo signs disappeared in all 18 patients who were diagnosed with GCA within a mean of 22 days after initiation of corticosteroid treatment. In nine of these patients, halos were not detectable at the 2-week time point, whereas in the remaining patients, halos disappeared at the 4-week time point. Further examinations were unremarkable. The halo was visible in the patient with Wegener's granulomatosis for 2 months after the initiation of treatment without any significant change in echogenity and disappeared thereafter. Finally, the halo sign in the patient with tuberculosis was reproducible at the first, but not at the second, follow-up examination.

## Discussion

A careful physical examination, including palpation of the temporal arteries, accompanied by an accurate medical history and laboratory data are all imperative for the diagnosis of GCA [12,16]. Moreover, clinicians should be familiar with the atypical presentation of the disease, including fever of unknown origin, respiratory tract symptoms, and large artery involvement [1]. Symptoms such as jaw claudication and diplopia significantly increase the probability to diagnose GCA [16,17]. In our prospective study, visual disorders and jaw claudication at baseline were associated with high specificity, albeit with low sensitivity. Although jaw claudication had 100% specificity, only three of our 22 patients with GCA reported this symptom. This frequency is clearly lower than expected [1-3], but similar findings have been reported in other cohorts of patients with GCA [18].

Accumulated evidence suggests that of greater sensitivity than clinical evaluation of patients are findings demonstrated by CDS of the temporal arteries [9-11,18,19]; CDS is of additive value in assessing the peripheral involvement of the disease [20] or in diagnosing concomitant GCA in patients with polymyalgia rheumatica [21]. Despite the fact that temporal artery biopsy is considered the gold standard for the diagnosis of GCA, patients with definite GCA frequently may have a negative biopsy [2,6,8]. In addition, the need for a large arterial segment (2 to 3 cm) due to the segmental nature of the vascular inflammation [22-25] and the practical difficulties in repeating it, make temporal artery biopsy not a simple procedure [26]. The present study was designed to specifically examine the diagnostic value of CDS with respect to common problems in the approach of a patient with clinically suspected GCA, for whom the decision for immediate treatment is imperative.

In agreement with previous studies [18,19], we found that the ultrasonographic evidence of a halo around the temporal artery lumen yields a fair sensitivity (82%) and a high specificity (91%) for the diagnosis of GCA. Although Salvarani *et al*. reported that only halos greater than 1 mm in thickness increased the probability of a positive biopsy result [15], our findings suggest that even small halos of 0.7 mm may predict GCA. Moreover, bilateral halos were evident in almost one third of patients with GCA, and the specificity of this finding for GCA diagnosis reached 100%, suggesting that in a given patient with clinically suspected GCA and bilateral halo signs, a temporal artery biopsy is not necessary. To the best of our knowledge, the specificity of this particular sign in CDS has not been examined previously.

It is well known that CDS is an investigator-dependent test in which the skill and the experience of the operator are important variables in determining the diagnostic accuracy of the method. For example, if colour setting is not properly selected, the arterial representation is not optimal and a thin halo may be missed in case colour covers both the artery wall and the lumen (that is, 'colour bleeding', false-negative finding). On the other hand, if colour covers only the centre of the artery, the periphery of the artery appears dark and may be misdiagnosed as a halo (false-positive finding). A formal evaluation of inter-reader variability was not conducted in our study; such data could help in minimizing false-positive or -negative findings which can be obtained even by experienced sonographers. Along this line, although a true positive finding cannot be ruled out in our patient with tuberculosis, the halo that was demonstrated in this particular case may represent a low examination quality. Moreover, the examination should be performed by a modern scanner with adequate resolution to investigate the small temporal arteries placed near the skin surface [10]. In agreement with previous reports [9,27], halos disappeared within a mean of 22 days after the initiation of corticosteroid treatment in our patients. The true interval for halo disappearance could be even shorter if closer follow-up points had been chosen. It should be noted, however, that directional biopsies performed in patients with unilateral halos may have partly influenced this result. Finally, we noticed that in four patients in whom GCA relapsed during tapering of corticosteroid treatment, halos reappeared and regressed again when the corticosteroid doses increased. Thus, although not systematically studied, CDS may be of significant value also in cases of GCA relapses.

We also found that directional temporal artery biopsies in all patients with halos and GCA were always positive, indicating that CDS examination before performing a biopsy could avoid 'generous' excisions of the temporal artery in many cases. Previous studies comparing CDS and histology of the temporal artery in GCA suggested that patients with halos had a more pronounced inflammatory cell infiltration in biopsy whereas patients without halos demonstrated histological signs of subtle inflammation [28]. A formal evaluation of the diagnostic value of the temporal artery biopsy alone, as well as a comparative analysis of CDS and biopsy findings, is not possible given that 20 of 49 biopsies were directed and 18 of them were positive for GCA, thus biasing any comparison. Regarding the presence of stenoses and occlusions in patients with GCA, the results reported here are in contrast to those of Schmidt and colleagues [9]. Blood-flow abnormalities were common in GCA and non-GCA patients, as well as in elderly healthy subjects and patients with macrovascular disease associated with diabetes mellitus or stroke (Table 2), suggesting that the presence of stenoses and occlusions of the temporal arteries does not increase the diagnostic value of CDS for GCA, at least in our hands. In a recent meta-analysis examining the results of 23 relevant studies, it was concluded that although CDS is a relatively accurate diagnostic method, cautious interpretation of the test results in terms of clinical manifestations and pretest likelihood is essential [29]. However, these conclusions are based on the combined analysis of any kind of abnormality found in CDS in patients with clinically suspected GCA, including perfusion and blood-flow abnormalities, and the diagnostic value of bilateral halo signs was not examined [29]. Moreover, all relevant studies have used the ACR classification criteria for GCA [4] as the reference standard for CDS. Such comparisons may be biased because these criteria have been developed for patients with vasculitis, whereas in the clinical setting a given elderly patient with suspected GCA may suffer from infectious or neoplastic diseases, as was the case in up to one third of the patients in our cohort.

**Figure 5:**
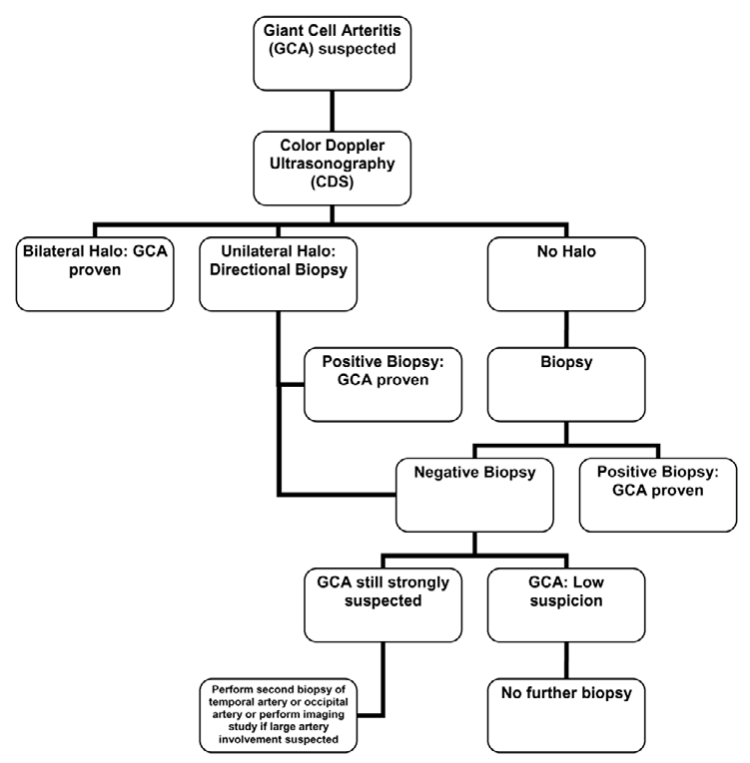
Proposed algorithm for the approach to diagnose giant cell arteritis (GCA) (modified from Hellmann and Hunder [3]).

Finally, our results may allow us to partially modify an established algorithm for the approach to diagnosing GCA, introduced by Hellmann and Hunder [3]. As depicted in Figure 5, we propose that after a careful clinical examination and assessment of relevant laboratory data, CDS examination should precede the biopsy in patients with suspected GCA. Among the various abnormalities that can be found in CDS, only the halo sign should be considered. In case of bilateral halo signs, treatment should be initiated without proceeding with biopsy. If unilateral halos are present, a decision of directional biopsy is justified. If the histology result is negative, clinicians should follow the algorithm introduced by Hellman and Hunder [3].

## Conclusion

CDS examination is an inexpensive, non-invasive, reproducible, and easy-to-perform method that should precede temporal artery biopsy in all patients with suspected GCA. Biopsy is not necessary in a substantial proportion of patients in whom bilateral halo signs around the temporal arterial wall are found in CDS. The presence of a unilateral halo allows a directional biopsy that has an increased probability to confirm the clinical diagnosis. Further studies to assess the test performance of CDS in assessing relapses of GCA are warranted.

## Abbreviations

ACR = American College of Rheumatology; CDS = colour duplex ultrasonography; CI = confidence interval; ESR = erythrocyte sedimentation rate; GCA = giant cell arteritis.

## Competing interests

The authors declare that they have no competing interests.

## Authors' contributions

M Karahaliou participated in the design of the study, performed CDS, and helped to draft the manuscript. GV participated in the design and coordination of the study and the clinical follow-up. SP participated in the coordination of the study and performed CDS. M Kanakis helped in the clinical follow-up and in the analysis and interpretation of the data and helped to draft the manuscript. KR participated in the coordination of the study and performed CDS. PS conceived of the study, participated in its design and coordination and the clinical follow-up, and drafted the manuscript. All authors read and approved the final manuscript.

## Figures and Tables

**Table 1:** Baseline characteristics and final diagnoses in 55 patients with clinically suspected GCA

Findings	GCA (*n *= 22)	Miscellaneous diagnoses (*n *= 33)
Age 50 years or above	22	33
Elevated ESR	19	30
Fever	9	19
New onset of headache	14	10
Diffuse musculoskeletal pain	7	15
Jaw claudication	3	0
Temporal artery tenderness	8	3
Acute visual impairement	5	2

CDS, colour duplex ultrasonography; ESR, erythrocyte sedimentation rate; GCA, giant cell arteritis.

**Table 2:** Abnormalities found in CDS of the temporal arteries in patients with clinically suspected GCA and control subjects

Final diagnoses (*n*)	Presence of halo (bilateral)	Blood-flow abnormalities
GCA (22)	18 (9)	9
Polymyalgia rheumatica (12)	0	4
Neoplasms (8)	0	2
Infections (10)	2 (0)	1
Wegener's granulomatosis (1)	1 (0)	1
Behçet's disease (1)	0	0
Polyarteritis nodosa (1)	0	1
Controls (age-/gender-matched)		
Healthy (15)	0	5
Diabetes mellitus and/or stroke (15)	0	6

CDS, colour duplex ultrasonography; GCA, giant cell arteritis.

**Table 3:** Sensitivity and specificity of baseline clinicolaboratory findings for the diagnosis of GCA in 55 patients studied prospectively

Findings	Sensitivity (%)	Specificity (%)
Elevated ESR	86	9
Fever	41	42
New onset of headache	64	70
Diffuse musculoskeletal pain	32	55
Jaw claudication	14	100
Temporal artery tenderness	36	91
Acute visual impairement	23	94
Unilateral halo sign	82	91
Bilateral halo sign	41	100

ESR, erythrocyte sedimentation rate; GCA, giant cell arteritis.
